# Prehospital time indicators before and after the implementation of an electronic information management system (EIMS): a cross-sectional study

**DOI:** 10.1186/s12873-025-01418-2

**Published:** 2025-11-24

**Authors:** Vahid Ghanbari, Nemat Shikhi, Farank Jafaari, Nader Salari, Amin Hosseinian-Far

**Affiliations:** 1 Prehospital Emergency Care Department, School of Paramedic, Kermanshah University of Medical Sciences, Kermanshah, Iran; 2 Student Research Committee, Kermanshah University of Medical Sciences, Dolatabad Blv, Imam Hossin Highway, Kermanshah, Iran; 3 Department of Medical Surgical Nursing, School of Nursing and Midwifery, Kermanshah University of Medical Sciences, Kermanshah, Iran; 4 Department of Biostatistics, School of Health, Kermanshah University of Medical Sciences, Kermanshah, Iran; 5 Department of Business Analytics and Systems, University of Hertfordshire, Hatfield, UK

**Keywords:** Prehospital emergency service, Information management system, Electronic patient care report (ePCR), Time indicators, Response time

## Abstract

**Background:**

The prehospital emergency system faces significant challenges, including a lack of coordination, primarily due to poor communication of information. An electronic information management system (EIMS) was introduced in Iran to improve coordination.

**Objectives:**

This study aims to assess the impact of the system on the time indicators in prehospital emergency services in Kermanshah, Iran.

**Methods:**

In this retrospective cross-sectional study conducted in 2022, time indicators were compared for 900 PCRs: 450 from the paper-based system (2016–2017) and 450 from the electronic PCR system (2017–2018). The time indicatorsincluded dispatch, filed, and hospital time indicators. Data were analyzed using SPSS version 17.

**Results:**

The triage time (94.1 ± 40.3 s) and delay time (111.9 ± 58.8 s) were significantly longer than the standard times (90 and 75 s, respectively). In the paper-based system, response (8.07 ± 3.6 min), scene (16.3 ± 8.2 min), and transportation times (13.07 ± 8.3 min) were shorter, than those in the EIMS: response (11.0 ± 6.3 min), scene (17.9 ± 9.3 min), and transportation (16.7 ± 12.06 min) times in EIMS (*p* < .05). However, other times indicators were significantly shorter in the EIMS compared to the paper-based system (*p* < .05).

**Conclusions:**

Except for triage and delay indices, all time indicators in both systems were significantly lower than the standard indicators. Implementation of the EIMS may face several technical and organizational challenges. It is important that these challenges be carefully considered.

**Supplementary Information:**

The online version contains supplementary material available at 10.1186/s12873-025-01418-2.

## Background

Emergency Medical Services (EMS) is a critical component of the healthcare system, which provides urgent prehospital care [1, 2]. The EMS system aims to deliver timely, life-saving interventions [3–5]. Timely access to information is critical for effective EMS operations [2, 3, 6], and integrating information technologies can improve communication, efficiency, and response times, thereby reducing costs and preventable deaths [3, 4, 7–9].

Despite the important role of timely access to information in the EMS system [10], the adoption of information and communication technologies (ICT) is slower than in other parts of healthcare systems [4, 8]. Previous studies have shown that inappropriate hardware, software, and organizational barriers hinder ICT implementation in emergency services [2, 6, 11], which can negatively affect time indicators [9]. A one-minute delay in treatment could reduce patient survival by 10% [7]. To address this, EMS agencies are adopting a variety of information system technologies to improve management and performance [2]. Technology integration improves prehospital care through features such as caller identification, location tracking, unit status monitoring, response optimization, and wireless dispatch [3, 12].

Gaeeni et al. (2021) reported that implementing an electronic information management system (EIMS) in Qom, Iran, significantly reduced ambulance response time [13]. Similarly, Afzali et al. found that EIMS improved service speed and EMT readiness in Kerman [14]. Al-Haliq et al. (2022) demonstrated that the majority of Emergency Medical Technicians (EMTs) perceive electronic Patient Care Reporting (ePCR) systems as beneficial for documentation [8]. Furthermore, Jensen et al. (2021) reported that electronic patient care records (ePCR) could enhance communication between EMTs, patients, and emergency department staff [15].

Despite benefits, technical, organizational, and usability issues can hinder effective ePCR implementation [2, 9, 16]. This study aimed to examine the impact of EIMS implementation on prehospital emergency time indicators.

## Methods

This descriptive-analytical retrospective study was conducted in 2022 within the EMS system of Kermanshah Province, Iran. The sample size was calculated using the mean estimation formula of continuous variables with a 95% confidence level. A sample size of 900 was determined. (Appendix).

### Sampling

An equal number of PCR were selected from both the paper-based and electronic systems. Time indicators were extracted from 450 paper-based PCRs completed during 2016–2017 and from 450 mission PCRs in the EMS information management system (EIMS) during 2017–2018. During the last three months of 2016–17, both systems operated in parallel to support EMS staff transition and prevent disruptions in patient care. EMTs completed paper PCRs during missions and then filled in the ePCRs after mission completion.

Only missions conducted under normal operational conditions were included, while those with missing time recordings were excluded. Any selected PCR that did not meet the inclusion criteria was omitted. Then, the next PCR number was selected. Subsequent to this, the time indices were extracted from the corresponding file.

Samples were selected using proportional sampling. First, the monthly number of missions was determined and the monthly ratio of required PCRs was calculated. Next, missions were stratified by location and reason within each month, and the category-specific ratios were computed. PCRs were then randomly selected from each stratum. Any mission that did not meet the inclusion criteria was excluded and replaced by the next eligible PCRs. Selection of PCRs was based on managerial data that were recorded. Time indicators were subsequently extracted from the corresponding files.

The extracted data included all time indicators provided by the National EMS organisation, including dispatch time indicators (announce, triage, call-out), field time indicators (delay, response, scene, transfer times), and in-hospital time, mission completion time, and total run time. Data were then extracted using the form validated in the study of Gaeeni et al. [13].

Announce, triage, and call-out times were not recorded in the paper-based system; therefore, only EIMS data were compared with the corresponding standards. Since turnout times vary between day (60 s) and night (90 s), their average (75 s) was used as a single benchmark.

### Data analysis

The collected data were entered into SPSS version 17 for analysis. One-sample and independent-samples t-tests were then applied to compare time indicators within and between systems, as well as against the standards.

## Results

About two-thirds of missions in both systems involved male patients. Roughly 25% were traffic accidents (106 paper-based, 124 EIMS), and over 50% were at home (272 paper-based, 232 EIMS). Around two-thirds (344 of 450) occurred in urban areas (> 70%). There were no statistically significant differences between the two systems in terms of the reason, type, or location of mission (*p* > .05) (Table 1).

**Table 1:** Comparison of the type and location of missions conducted by prehospital emergency systems in the City of Kermanshah

Variable		EIMS^1^		Paper-based System		Test Statistics	P-Value
		Frequency	%	Frequency	%		
Gender	Female	172	38.3	161	35.7	0.127	0.529
	Male	277	61.7	290	64.3		
Main Reason at the Time of Call	Accident	124	27.6	106	23.5	10.66	0.058
	Chest pain	39	8.7	52	11.5		
	Shortness of breath	37	8.2	32	7.1		
	Poisoning	21	4.7	21	4.7		
	level of consciousness	69	15.4	48	10.6		
	Other	159	35.4	192	42.6		
Location of Emergency (Reported Location)	Residential	232	51.7	272	60.3	9.3	0.054
	Industrial	20	4.5	19	4.2		
	Traffic areas	161	35.9	133	29.5		
	Recreational places	22	4.9	21	4.7		
	Educational places	14	3.1	6	1.3		
Inside City/ Outside City	Urban	356	79.1	344	76.4	1.05	0.305
	Non-Urban	94	20.9	106	23.6		

EIMS: Electronic Information Management System

Table 2 compares the average time indicators in the electronic and paper-based systems with standard benchmarks using a one-sample t-test.

**Table 2:** Comparison of time indicators in electronic and Paper-based information management systems with standard benchmarks

Variables	Mean ± SD	Min	Max	Standard Time	T	*P*-value
Dispatch Time Indicators in Electronic Information System						
Announce Time	4.8 ± 2.06	1	30	8s^a^	-31.8	0.001
Triage time	94.1 ± 40.3	12	220	90s	2.1	0.031
Call-out	15.8 ± 14.1	5	118	60s	-65.1	0.001
Delay time	111.92 ± 58.8	17	364	75s	13.40	0.000
Response Time in Urban Missions						
Paper	8.07 ± 3.6	2.30	30.38	12 M^b^	-19.91	0.000
Electronic	11.02 ± 6.3	1	50		-2.91	0.004
Response Time in Out_of_urban Missions						
Paper	8.6 ± 4.7	2.45	37.20	14 M	11.4	0.001
Electronic	10.7 ± 8.07	1	68		-3.8	0.001
Scene time						
Paper	16.3 ± 8.2	1	63.3	20 M	-9.6	0.001
Electronic	17.98 ± 9.31	1	70		-4.6	0.000
Inhospital						
Paper	19.05 ± 10.04	1	74	15 M	8.4	0.001
Electronic	10.5 ± 9.2	1	119		-21.8	0.001

^*a*^*S*: Second, ^*b*^*M: Minute*

The mean announcement time for the EIMS was 4.8 ± 2.0 s, significantly faster than the standard 8 s (*p* = .001). However, triage time (94.1 ± 40.3 s) and delay time (111.9 ± 58.8 s) were both significantly longer than the standard times of 90 and 75 s, respectively (*p* = .03).

Response times for in-city missions were significantly shorter in both systems (paper-based: 8.07 ± 3.6 min vs. electronic: 11.02 ± 6.3 min; (*p* = .001) compared to the standard benchmark. Out-of-city response times also remained below 14 min (paper-based: 8.6 ± 4.7 min; electronic: 10.7 ± 8.07 min) (*p* = .001).

The average scene time was shorter than the standard 20 min in both systems—16.3 min for paper-based and 17.98 min for electronic—and this difference was statistically significant (*p* = .001) (Table 2). The in-hospital time was significantly longer in the paper-based system (19.05 ± 10.04 min) compared to the standard 15 min (*p* = .001). Conversely, the electronic system’s in-hospital time (10.5 ± 9.2 min) was significantly less than the standard (*p* = .001) (Table 2).

Table 3 presents a comparison of time indicators between the electronic and paper-based information management systems using the Student’s t-test.

**Table 3:** Comparison of average time indicators in electronic and Paper-based information management systems

Variables	EIMS (Mean ± SD)	Paper-based (Mean ± SD)	T	*P*-value
Urban Response Time	11.02 ± 6.3	8.07 ± 3.6	7.4	0.001
Out-of-City response Time	10.8 ± 8.05	8.6 ± 4.7	2.2	0.030
Scene Time	17.98 ± 9.31	16.2 ± 8.2	1.5	0.127
Transportation Time	16.7 ± 12.06	13.07 ± 8.3	5.3	0.001
Inhospital Time	10.5 ± 9.2	19.05 ± 10.04	-13.2	0.001
Completion of Mission	9.5 ± 12.8	14.99 ± 16.91	-5.4	0.001
Total Run Time	67.8 ± 25.2	73.8 ± 25.7	-3.4	0.001

As shown in Table 3, response times for both urban and out-of-city missions were significantly shorter in the paper-based system (8.07 ± 3.6 min) than in the electronic system (11.02 ± 6.3 min) (*p* = .03). There was no significant difference in scene time between the two systems (*p* = .127). Transportation time was also shorter in the paper-based system (13.07 ± 8.3 min) versus the electronic system (16.7 ± 12.06 min). However, in-hospital time, mission completion, and total run time were all significantly longer in the paper-based system (*p* = .01) (Table 3).

## Discussion

This study compared prehospital emergency time indicators before and after implementing the EIMS in Kermanshah’s emergency services in 2022. Most time indicators in both systems were better than the standard times. While the EIMS improved post-mission times, response and scene times did not show significant improvements with its implementation.

Traffic accidents were the most common reason for dispatch, accounting for 27% of cases. Zeraatchi et al. (2018) identified trauma as the leading cause of emergency missions [17] and Ranjbar et al. found that 42% of EMS missions were trauma-related [18]. These findings likely reflect Iran’s unique epidemiological context, characterized by a high trauma incidence and conservative telephone triage protocols that require ambulance response to all reported accidents with casualties, placing substantial demand on the system.

Our study showed announce times (4.8 ± 2.06 s) and call-out times (15.8 ± 14.1 s) were significantly shorter in the EIMS compared to standard times (8 and 60 s). This likely results from computer-assisted technology enabling automatic number recognition and EMS unit verification. However, telephone triage time in the EIMS (94.1 s) was significantly longer than the standard (90 s, *P* = .01), possibly due to more thorough data collection via call recording, leading dispatchers to gather detailed information for accurate prioritization. Landman et al. similarly found that dispatch times may initially increase until staff fully adapt to ePCR in prehospital settings [19]. Further research should explore causes of prolonged triage times.

Surprisingly, our study found that delay time in the EIMS (111.92 ± 58.8 s) exceeded the standard time (75 s). This aligns with Feizollahzadeh et al. (2022) and Chegini et al. (2024), who reported delay times around 150 s [20, 21]. In contrast, Asadi et al. (2021) observed a delay time of about 60 s in Ardabil [22]. Khorasani-Zavareh et al. (2018) shown that = EMS workload, station design, mission timing, responder activities, and use of personal protective equipment (PPE) may affect delay time time [23]. Jasbi et al. (2021) also stated that healthcare organizations experiencing uncertain situation in migration to ePCR [9]. Moreover, Complexity in designing ePCR and subdividing it into multiple pages can hinder first-time users from utilizing the system efficiently [2, 16]. So, future research should explore the effect of staff training adequacy, system usability on delay time.

Our study found that response times were shorter with the paper-based system (in city: 8.07 min; out of city: 8.6 min) compared to the EIMS (both in and out of city: 11.02 min), a statistically significant difference (*p* = .03). This contradicts earlier studies reporting reduced response times with EIMS implementation [9, 13, 14]. One possible explanation for the longer ePCR response time is technical issues such as low battery or frozen screens. These issues hinder access to patient information or the mission address, causing the response time to lengthen [16].

The result also showed that, in both information management systems, urban and rural response times were shorter than the standard 12 min. Ranjbar et al. (2021) reported response times of 11.30 and 8.43 min for paper-based and electronic systems, respectively [18], while Asadi et al. found a mean response time of 7 min [22]. Factors affecting response times include traffic, building and population density, and emergency base accessibility. In Kermanshah, relatively low traffic outside peak hours and lower building density compared to cities like Tehran likely contributed to the shorter response times observed.

This study found transfer time was significantly shorter in the paper-based system (13.07 min) than in the EIMS (16.07 min). In contrast, Gaeeni et al. (2020) reported no significant difference between the systems [13]. Longer transfer times with EIMS may result from more precise ambulance tracking via GPS, while inaccurate recording challenges in paper-based systems may explain their shorter times [13]. Moreover, Altuwaijri et al. (2018) reported that EMTs perceived paper-based PCR as concise with important information highlighted. In contrast, ePCR offers more fields of information subdivided across multiple pages, making navigation time-consuming or EMTs lacking training in filling the required information [16].

Statistical analysis showed that mean in-hospital time with the EIMS (10.5 min) was significantly shorter than both the standard time (15 min) and the paper-based system (19.05 min) (*p* = .01). In contrast, in-hospital time in the paper-based system was significantly longer than the standard time (*P* = .01). This aligns with Gaeeni et al. (2021), who reported shorter hospital times after EIMS adoption (9.2 vs. 12.57 min with paper-based systems) [13]. Similarly, Anantharaman and Swee Han (2001) found that an internet-based information system reduced hospital stay from 15 to 8 min [24]. The EIMS likely improves data transmission speed and accuracy, facilitating better preparation—such as bed assignment and staff readiness—leading to reduced patient handover times. Afzali et al. also reported decreased hospital stay times with EIMS adoption [14].

The time from patient handover to return to station was significantly shorter with the EIMS (9.5 min) compared to the paper-based system (14.99 min) (*p* = .001). EIMS implementation improves information exchange and reduces patient service times [3, 14]. Additionally, ambulance GPS tracking in EIMS allowed dispatchers to monitor locations, contributing to the notably shorter ‘handover to mission completion’ time compared to the paper-based system.

The total run time with EIMS (67.8 min) was significantly shorter than with the paper-based system (73.8 min). Feizollahzadeh et al. (2022) reported a total run time of 61.5 min [21], while Asadi et al. (2021) found 52.5 min [22]. These differences may stem from variations in emergency base density, regional traffic conditions, and city-specific factors. Moreover, ambulance location tracking in EIMS allows EMS technicians to promptly report mission completion, contributing to shorter total mission times compared to the paper-based system, where reporting may be delayed or missed.

### Limitation

Since this study was conducted retrospectively, there is a possibility of recording errors, particularly in the paper-based system, which may affect data accuracy. Ease of use is a key factor in computer-assisted programs, and an initial implementation of the system may present challenges that affect time indicators. Furthermore, a three-month transition period may have been insufficient for staff to fully adapt to the new system, potentially impacting time indicators.

## Conclusion

The study found that, with the exception of telephone triage and delay times, most time indicators within the electronic information management system (EIMS) were shorter than the standard. The EIMS improved post-arrival metrics but did not significantly affect pre-arrival times. These findings suggest that, with real-time monitoring and accurate data recording, the EIMS can enhance prehospital emergency performance. Moreover, our results indicate that the implementation of electronic patient care reporting (ePCR), as anticipated, faced challenges that may limit EMS performance gains. Consequently, EMS managers should carefully consider these challenges during the implementation of new systems. Future studies should investigate the possible reasons for longer triage, response, and delay time indicators within the EIMS.

## Supplementary Information

Below is the link to the electronic supplementary material.

Supplementary Material 1

## Data Availability

The datasets generated and/or analyzed during the current study are available from the corresponding author on reasonable request.

## Abbreviations

EMS: Emergency Medical Services
EIMS: Electronic Information Management System
EMT: Emergency Medical Technicians
PCR: Patient Care Reports
