# Supplementary material for: Prehospital time indicators before and after the implementation of an electronic information management system (EIMS): a cross-sectional study
Source: BMC Emerg Med. 2025 Nov 24;25:264. doi: 10.1186/s12873-025-01418-2 (PMC12751553; doi:10.1186/s12873-025-01418-2)
Supplement: Supplementary file 1 — Supplementary Material 1 [file 12873_2025_1418_MOESM1_ESM.docx]

Sample size parameters and Formula

n = (Zα/2)² * (σ)² / d².

α=0.05

Zα/2=1.96,

σ=5

d=0.03

n=(1.96)² *(5)²/(0.03)² n=900
